# Biochemical and Structural Analysis of a Novel Esterase from *Caulobacter crescentus* related to Penicillin-Binding Protein (PBP)

**DOI:** 10.1038/srep37978

**Published:** 2016-12-01

**Authors:** Bum Han Ryu, Tri Duc Ngo, Wanki Yoo, Sojeong Lee, Boo-Young Kim, Euijoo Lee, Kyeong Kyu Kim, T. Doohun Kim

**Affiliations:** 1 Department of Chemistry, College of Natural Science, Sookmyung Women’s University, Seoul 140-742, Korea; 2 Department of Molecular Cell Biology, Samsung Biomedical Research Institute, Sungkyunkwan University School of Medicine, Suwon, 440-746, Korea

## Abstract

Considering that the prevalence of antibiotic-resistant pathogenic bacteria is largely increasing, a thorough understanding of penicillin-binding proteins (PBPs) is of great importance and crucial significance because this enzyme family is a main target of β-lactam-based antibiotics. In this work, combining biochemical and structural analysis, we present new findings that provide novel insights into PBPs. Here, a novel PBP homologue (CcEstA) from *Caulobacter crescentus* CB15 was characterized using native-PAGE, mass spectrometry, gel filtration, CD spectroscopy, fluorescence, reaction kinetics, and enzyme assays toward various substrates including nitrocefin. Furthermore, the crystal structure of CcEstA was determined at a 1.9 Å resolution. Structural analyses showed that CcEstA has two domains: a large α/β domain and a small α-helix domain. A nucleophilic serine (Ser^68^) residue is located in a hydrophobic groove between the two domains along with other catalytic residues (Lys^71^ and Try^157^). Two large flexible loops (UL and LL) of CcEstA are proposed to be involved in the binding of incoming substrates. In conclusion, CcEstA could be described as a paralog of the group that contains PBPs and β-lactamases. Therefore, this study could provide new structural and functional insights into the understanding this protein family.

The bacterial cell wall is composed of polymeric glycan chains with alternating N-acetylglucosamine (NAG) and N-acetylmuramic acid (NAM), which are crosslinked by short peptide branches^1^,^2^,^3^. This peptidoglycan layer forms the major structural component of the protective barrier and disruption of these layer leads to bacterial cell lysis. Penicillin-binding proteins (PBPs) are a large group of periplasmic enzymes that catalyze essential functions in the synthesis, modification, and maintenance of these peptidoglycans^4^,^5^. PBPs are responsible for two unique enzymatic activities; transglycosylation to form the glycan backbone by polymerizing disaccharides, and transpeptidation to catalyze cross-linking between adjacent glycan chains to create a mesh-like structure. In addition, PBPs are the targets of β-lactam antibiotics including penicillin and cephalosporin, which bind to the active site of PBPs as structural homologs of D-alanyl-D-alanine^6^,^7^.

Penicillin-binding proteins (PBPs) are classified into high-molecular-weight (HMW) PBPs and low-molecular-weight (LMW) PBPs based on molecular weight and sequence homology. HMW PBPs are responsible for peptidoglycan polymerization, cross-linking, and insertion of the peptidoglycan precursors into the preexisting strands through transglycosylation and transpeptidation reactions. HMW PBPs can catalyze both the polymerization of a peptidoglycan from disaccharide peptides (transglycosylase) and the cross-linking of muramyl peptides (transpeptidase). HMW PBPs are divided into subclass A and subclass B, which are bifunctional transpeptidases/transglycosylases (subclass A) and monofunctional transpeptidases (subclass B), respectively. The low-molecular-weight (LMW) PBPs have only the transpeptidase (TP) domain that catalyzes carboxypeptidase and endopeptidase activity *in vitro*, which play a role in peptidoglycan maturation.

*Caulobacter crescentus*, an important prokaryote for studying cellular differentiation and asymmetric division, is a widespread Gram-negative bacterium that has two distinct life stages; a motile swarmer cell and a sessile stalked cell^8^,^9^. Recently, it has been shown that PBPs in *C. crescentus* can interact with the cell wall synthetic complexes for cell elongation or stalk growth, facilitating the independent regulation of distinct growth processes^10^,^11^. However, biochemical studies of PBPs or their homologs in this microbe are largely unknown, and our understanding about their molecular structures and catalytic mechanism is still limited.

Recently, a number of bacterial enzymes with high similarities to PBPs have been identified from metagenomics libraries. These include EstU1^12^, Est22^13^, EstM-N1^14^, and EstC^15^. In a previous study, identification and preliminary X-Ray diffraction analysis of a novel PBP homolog (CcEstA, CCNA_00255) in *Caulobacter crescentus* CB15 were reported^16^. CcEstA was shown to catalyze the hydrolysis of industrially important compounds including ketoprofen ethyl ester. Here, biochemical and structural analysis of CcEstA were performed, which could pave a way for understanding the structure and function of PBP family proteins.

## Results and Discussion

### Sequence analysis of CcEstA

**Figure 1:**
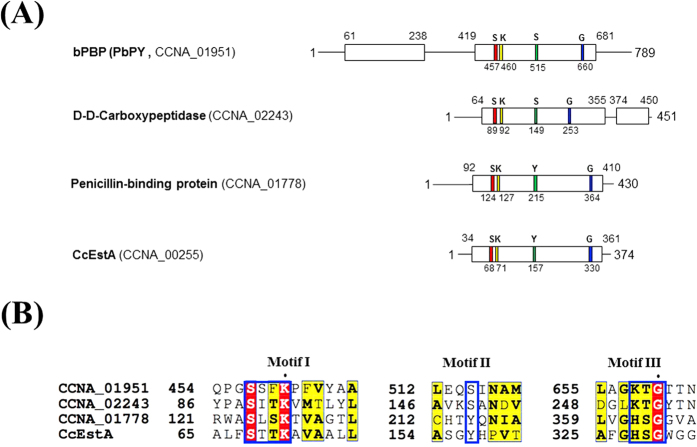
Overall structural organization and multiple sequence alignment of CcEstA. (**A**) The structural organization of CcEstA is compared with other related proteins in *Caulobacter crescentus*. Note that important catalytic residues (colored residues) are highly conserved and lie on spatially similar positions in each protein. (**B**) Multiple sequence alignment of CcEstA was performed with related proteins in *C. crescentus* using *Clustal Omega* and *ESPript*. Only three characteristic motifs of these proteins (motif I, II and III) are shown for clarity and highly conserved residues are shown in red.

The primary sequence of CcEstA is composed of a single polypeptide chain with 374 amino acids. Sequence analysis showed that CcEstA shared some sequence identity with D-Ala-D-Ala transpeptidase from *Streptomyces sp.* R61 (16.5%, P15555), and *Bacillus halodurans* (14.5%, P32959), and with β-lactamases from *Ochrobactrum anthropi* SV3 (14.0%, Q9ZBA9) and *Enterobacter cloacae* (16.0%, P05364). In protein databases, similar sequences to CcEstA were mainly annotated as members of the PBP or transpeptidase (TP) superfamilies. All PBPs or their homologs are believed to contain three characteristic motifs (Motifs I, II, and III), which are involved in catalysis^4^,^6^,^17^. These motifs are highly conserved with similar spacing along the polypeptides, which suggests a common evolutionary origin^18^,^19^. In addition, similar motifs and residues are also found in DD-peptidase^20^ and D-amino acid amidase^21^. Three sequence motifs (motif I, II, and III) of CcEstA have been identified through sequence comparisons with other PBP family proteins. Motif I typically includes a catalytic serine residue in the tetra-peptide of SxxK. In the case of CcEstA, a nucleophilic Ser^68^ is indeed found in the characteristic tetrapeptide, S^68^-x-x-K^71^. In this motif, Ser acts as a nucleophile, while Lys is involved in the acylation step as a general base. The key residue of motif II is a Tyr or Ser, the hydroxyl group of which is involved in activating and deacylating the nucleophilic Ser^7^. Interestingly, unlike other two motifs, motif III is not strictly conserved in the PBP family or its homolog proteins. In the case of CcEstA, His^328^-Ser^329^-Gly^330^ is found in motif III, whereas other closely-related proteins contains highly conserved Lys(K)-Thr(T)-Gly(G) motif at the same position. Therefore, only a glycine among these three residues is conserved. As shown in Fig. 1, CcEstA has similar catalytic residues to the TP domains in bifunctional penicillin-binding protein (PbpY, CCNA_01951), DD-carboxypeptidase (CCNA_02243), and penicillin binding protein (CCNA_01778) in the chromosome of *Caulobacter crescentus*. Taken together, multiple sequence alignment suggests that CcEstA shares sequence similarity with PBPs or the TP domain of bPBPs, suggesting that they all share common features in their functions and structures.

### Biochemical characterization of CcEstA

**Figure 2:**
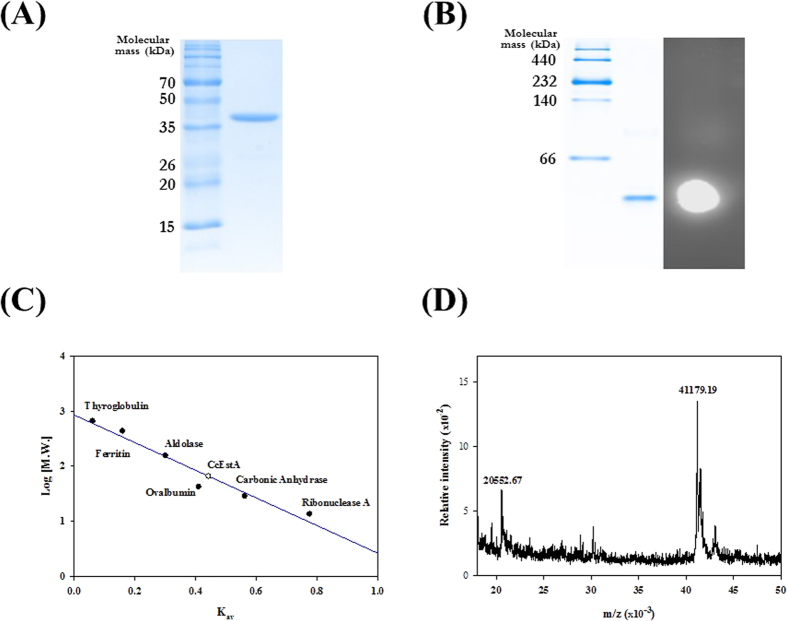
Characterization of CcEstA. (**A**) Purified CcEstA was analyzed by SDS-PAGE and then stained with Coomassie Brilliant Blue (*left*; molecular standards marker, *right*; purified CcEstA). (**B**) Native-PAGE analysis with 4-MU overlay assay of CcEstA. Only one band appears on Native-PAGE gel (*left*) showing the fluorescent signal from 4-MU catalysis (*right*). (**C**) Size exclusion chromatography of CcEstA under non-denaturing conditions. (**D**) MALDI-TOF mass spectrum of CcEstA. The [M+H]^+^ and [M+2 H]^2+^ ion peaks were found at m/z value of 20,552, and 41,179, respectively.

The recombinant CcEstA was purified to near-homogeneity, and a single band with a molecular mass of ~37 kDa was observed after Coomassie brilliant blue (CBB) staining (Fig. 2A). An overlay activity assay with 4-methylumbelliferyl (4-MU) acetate as a substrate showed high fluorescence due to the formation of 4-methylumbelliferone in native PAGE^22^,^23^. In zymogram analysis, the molecular mass of active CcEstA is smaller than 66 kDa, implying that CcEstA is monomeric conformation (Fig. 2B). A similar behavior indicating a monomeric state in solution was also observed in size exclusion chromatography (Fig. 2C). Therefore, CcEstA seems to function as a monomer, which is consistent with other recently reported PBP structures^24^,^25^,^26^. This is further supported by the fact that there are no expected dimeric interfaces within CcEstA as determined by the PISA program^27^. Furthermore, Maldi-tof mass spectrometric analysis showed a major peak (m/z) at 41.1 kDa, which is consistent with the calculated molecular mass of recombinant CcEstA (Fig. 2D).

**Figure 3:**
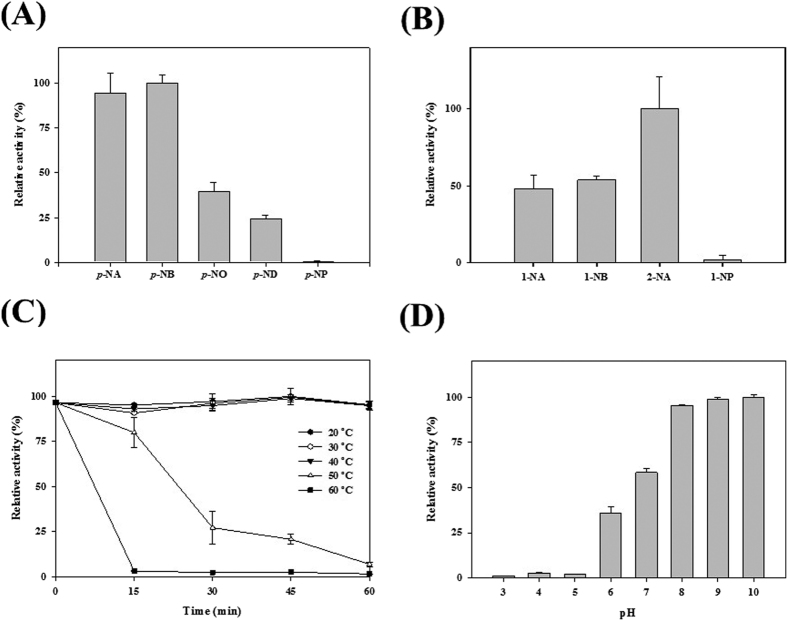
Enzyme assays of CcEstA. (**A**) Substrate-specificity assay of CcEstA using *p*-nitrophenyl esters with different acyl chain lengths. (**B**) Regioselectivity assay of CcEstA using naphthyl derivatives. (**C**) Activity of CcEstA in various temperatures from 20 to 60 °C for 1 h. (**D**) Activity of CcEstA in different pH solutions from pH 3 to 10.

The substrate specificity of CcEstA was assessed using *p*-nitrophenyl esters with different acyl chain lengths^28^. As shown in Fig. 3A, CcEstA showed a strong preference toward short-chain substrates such as *p*-nitrophenyl acetate (*p*-NA; C2) and *p*-nitrophenyl butyrate (*p*-NB; C4). However, less than 30% of initial enzyme activity was detected when longer chain substrates of *p*-nitrophenyl octanoate (*p*-NO; C8) or *p*-nitrophenyl decanoate (*p*-ND; C10) were used. No enzyme activity was noticed toward *p*-nitrophenyl phosphate (*p*-NP). Similarly, EstU1, Est22, EstM-N1 or EstC showed substrate preference for *p*-nitrophenyl butyrate (C4), while no enzyme activities of these enzymes was observed for *p*-nitrophenyl esters with longer chains (from C12 to C18)^12^,^13^,^14^,^15^. When diverse naphthyl derivatives were used as substrates, the highest activities were obtained with 2-naphthyl acetate (2-NA), followed by 1-naphthyl acetate (1-NA), and 1-naphthyl butyrate (1-NB). In contrast, CcEstA showed very little activity toward 1-naphthyl phosphate (1-NP). The deacetylation activity of CcEstA towards various acetylated substrates showed that the highest activities were obtained with 2-naphthyl acetate (2-NA), followed by *p*-nitrophenyl acetate (*p*-NA) (Fig. S4).

For assessing thermostability of CcEstA, residual activity during the incubation of CcEstA at temperature ranges at 20–60 °C was monitored at 15 min intervals for 60 min. As shown in Fig. 3C, CcEstA was very stable up to 40 °C. However, at 50 °C, CcEstA lost its activity very rapidly and retained only ~25% of initial activity after 30 min. No enzyme activity was observed at 60 °C. To determine the optimum pH for CcEstA, enzyme activity was monitored at pH values from 3.0 to 10.0 (Fig. 3D). Although CcEstA was unstable in acidic environments, it was highly stable at alkaline pH values from 8.0 to 10.0, retaining almost maximum activity. At pH 7.0, only ~50% of the initial activity at pH 9.0 was observed. This behavior was also observed in EstU1^12^ or EstM-N1^14^, which was highly stable at an alkaline pH of 9.0.

**Figure 4:**
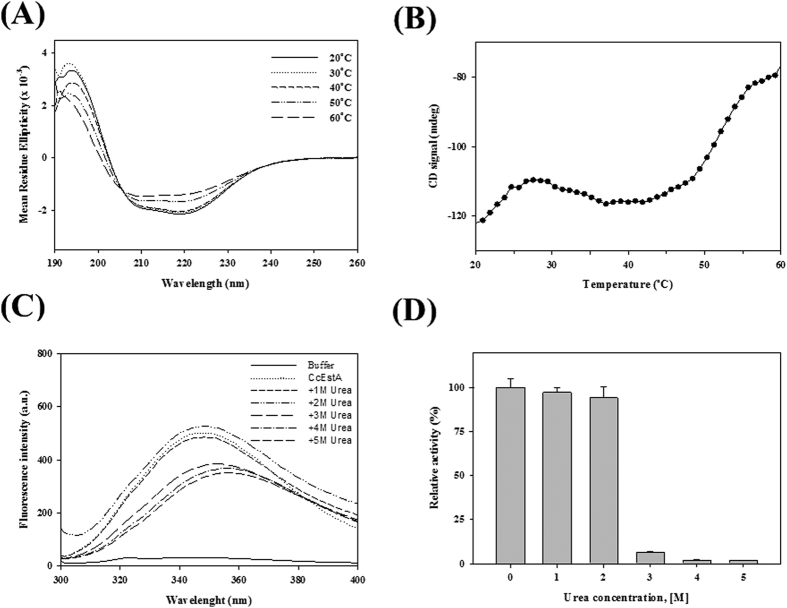
Structural stability of CcEstA. (**A**,**B**) Thermal analysis of CcEstA was monitored in various temperature from 20 to 60 °C using circular dichroism spectrometry (A; from 190 to 260 nm, B; at 222 nm). Note that CcEstA undergoes notable changes in structure above 50 °C. (**C**,**D**) Chemical unfolding of CcEstA. Intrinsic fluorescence spectra (**C**) and relative enzyme activity of CcEstA with various concentrations of urea from 0 to 5 M were recorded after 1 h incubation (**D**).

The structural stability of CcEstA in terms of thermal and chemical unfolding was investigated by circular dichroism (CD) and intrinsic fluorescence analysis, respectively^29^. Subsequent far-UV CD spectra analysis revealed that CcEstA retains its stable conformation up to 40 °C without any significant structural changes. Above 50 °C, CD spectra showed increased values from 210 to 230 nm region, which is consistent with the results of enzyme activity assays (Fig. 4A). To determine the melting point, CD signals of CcEstA were monitored at 222 nm in temperatures from 20 to 60 °C. Remarkable structural transitions were observed around 50 °C (Fig. 4B). Chemical unfolding of CcEstA was monitored upon the addition of urea as determined by changes in intrinsic fluorescence (Fig. 4C). Without urea, native CcEstA exhibited a λ_max_ of 349 nm and red shift of λ_max_ to 364 nm was observed with a significant decrease of fluorescence intensity after the addition of 3 M urea. Additionally, CcEstA showed very little activity at concentrations of 3 M urea and above (Fig. 4D).

**Figure 5:**
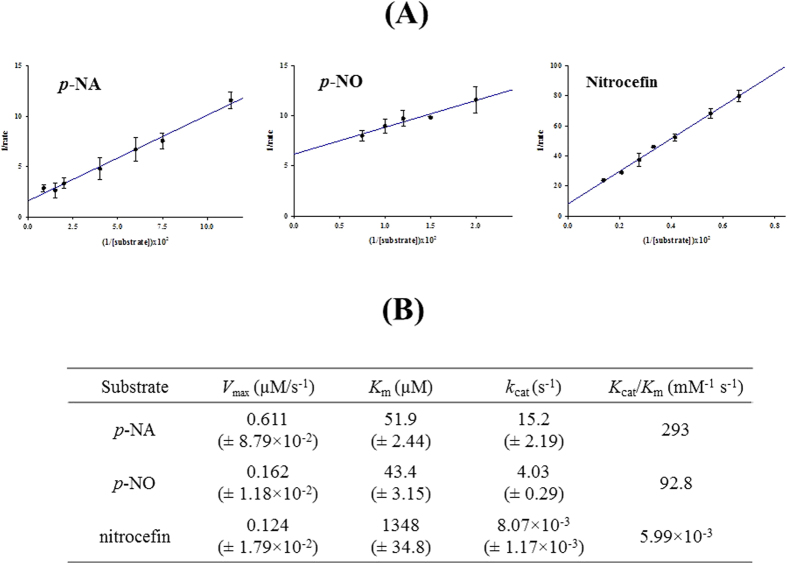
Kinetics analysis of CcEstA. With three different substrates (*p*-nitrophenyl acetate; *p*-NA, *p*-nitrophenyl octanoate; *p*-NO, and nitrocefin), V_max_, K_m_, and *k*_cat_ of CcEstA were determined from the Lineweaver-Burk plot. Standard errors for the parameters are shown in parenthesis.

As shown in Fig. 5A, CcEstA exhibited hyperbolic Michaelis-Menten kinetics for three different substrates (*p*-NA, *p*-NO, and nitrocefin). From the values of kinetic parameters, catalytic efficiency (*kcat*/Km) for *p*-NA hydrolysis is about 1.25-fold higher than observed for *p*-NO hydrolysis (Fig. 5B). Interestingly, CcEstA showed significantly different kinetic parameters for nitrocefin with approximately a 27-fold higher K_m_ (1348 μM) and a 1880-fold lower *kcat* (0.00807 s^−1^) compared to *p*-NA. Therefore, compared with the *p*-NA or *p*-NO, the catalytic efficiency for nitrocefin was very low, revealing that CcEstA is more efficient on *p*-nitrophenyl esters than nitrocefin.

**Figure 6:**
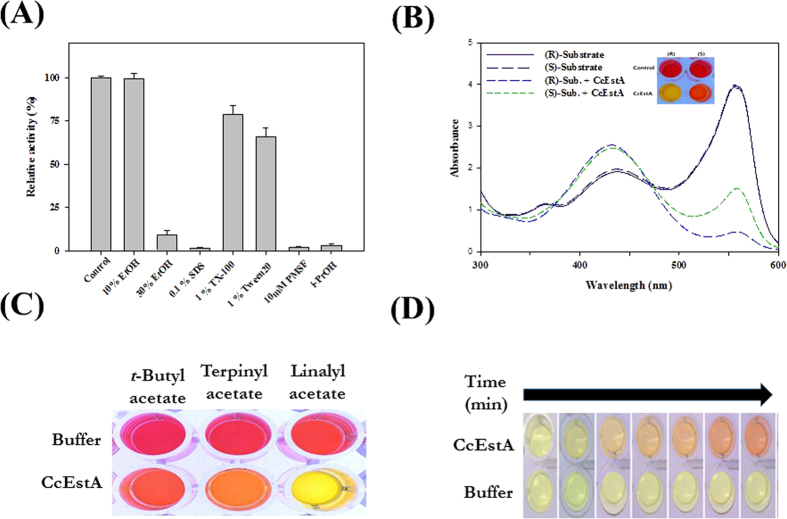
Biochemical analysis of CcEstA. (**A**) Chemical stabilities of CcEstA. The residual activities of CcEstA after 1 h incubation with different solvents are expressed relative to the initial activity (100%). (**B**) pH shift assay was performed to assess the enantioselectivity of CcEstA toward *(R)*- and *(S)*- methyl-3-hydroxy-2-methylpropionate. (**C**) Tertiary alcohol production by CcEstA using *tert*-butyl acetate, α-terpinyl acetate and linalyl acetate (LA). (**D**) β-lactamase activity of CcEstA using nitrocefin.

**Figure 7:**
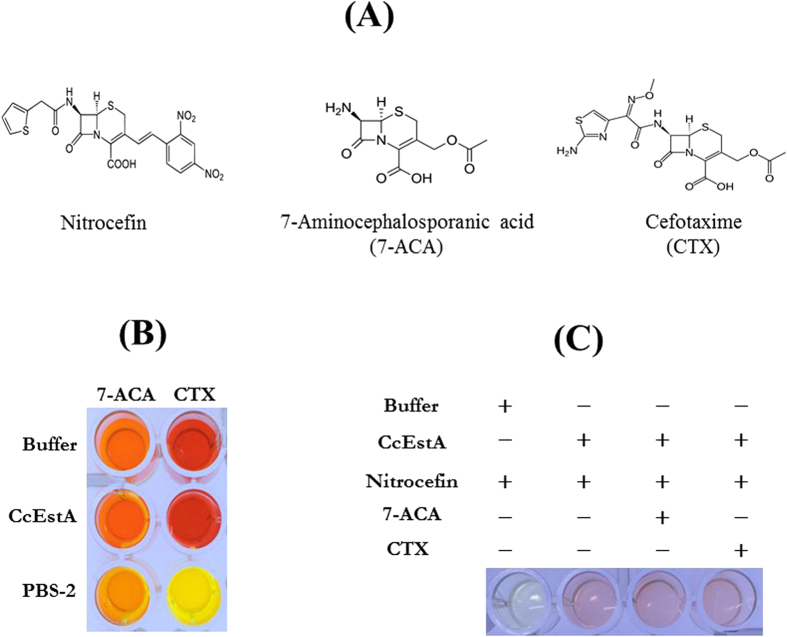
Hydrolytic activity of CcEstA toward β-lactam-related compounds. (**A**) Chemical structures of nitrocefin, 7-aminocephalosporanic acid (7-ACA), and cefotaxime (CTX). (**B**) A pH shift assay was conducted to verify the hydrolytic activity of CcEstA toward 7-ACA and CTX. PBS-2, a bacterial PBP homologue from *Paenibacillus sp.*, was used as a positive control^55^. Note that CcEstA showed almost no hydrolytic activity toward 7-ACA and CTX, while nitrocefin can be hydrolyzed. (**C**) Competitive inhibition assay of CcEstA. 7-ACA and CTX were used as competitive inhibitors of nitrocefin. Note that both 7-ACA and CTX have almost no inhibitory effect on nitrocefin hydrolysis.

The chemical stability of CcEstA was also assessed with various kinds of detergents and organic solvents. As shown in Fig. 6A, CcEstA was stable in 10% ethanol (EtOH) without any loss of activity. Furthermore, it could retain over 60% of its initial activity in 1% Triton X-100 (TX-100) or 1% Tween 20 (Tw-20). For enantioselectivity analysis, a pH-drop colorimetric assay was used with (*R*)- and (*S*)-methyl-β-hydroxyisobutyrate^30^. Following incubation with CcEstA, the reaction mixture containing the (*R*)-enantiomer turned yellow more rapidly, indicating the (*R*)-selectivity of CcEstA (Fig. 6B). The ability of CcEstA to hydrolyze tertiary alcohol esters (TAEs) was examined using *tert*-butyl acetate, α-terpinyl acetate, and linalyl acetate as substrates^28^. As shown in Fig. 6C, CcEstA hydrolyzed *tert*-butyl acetate, linalyl acetate, and the bulky α-terpinyl acetate. A pH-indicator-based colorimetric assay based upon the release of acetic acid was employed. As shown in Fig. 6C, CcEstA hydrolyzed *tert*-butyl acetate, α-terpinyl acetate, and linalyl acetate. Considering the fact that tertiary alcohols are expensive building blocks in the pharmaceutical industry^31^, CcEstA could be used as a biocatalyst for industrial applications. Additionally, to investigate the β-lactamase activity of CcEstA, a chromogenic β-lactamase substrate (nitrocefin) was used as a substrate^32^. As shown in Fig. 6D, CcEstA apparently showed β-lactamase activity toward nitrocefin, which was verified with the color change of the reaction mixture. The β-lactamase activity of CcEstA was further investigated using 7-ACA and CTX (Fig. 7A). Compared to PBS-2, CcEstA showed no catalytic activities towards 7-aminocephalosporanic acid (7-ACA) or cefotaxime (CTX) as determined by a pH shift assay (Fig. 7B). Furthermore, both 7-ACA and CTX had almost no inhibitory effects toward nitrocefin hydrolysis by CcEstA (Fig. 7C), implying that the substrate-binding site of CcEstA is not suitable for 7-ACA or CTX. In this respect, the absence of N in the S(Y)xN motif could be related to the relatively low activity of CcEstA towards β-lactam compounds because this N is usually employed in hydrogen bonding to β-lactam side chain amide groups^4^,^5^,^6^. In accordance with CcEstA, EstC and EstM-N1 have shown significant hydrolyzing activity for nitrocefin, but did not display activity for β-lactam substrates. However, EstU1 and Est22 were shown to possess a significant β-lactam hydrolytic activity for cephalosporin derivatives.

### Overall Structure of CcEstA

**Figure 8:**
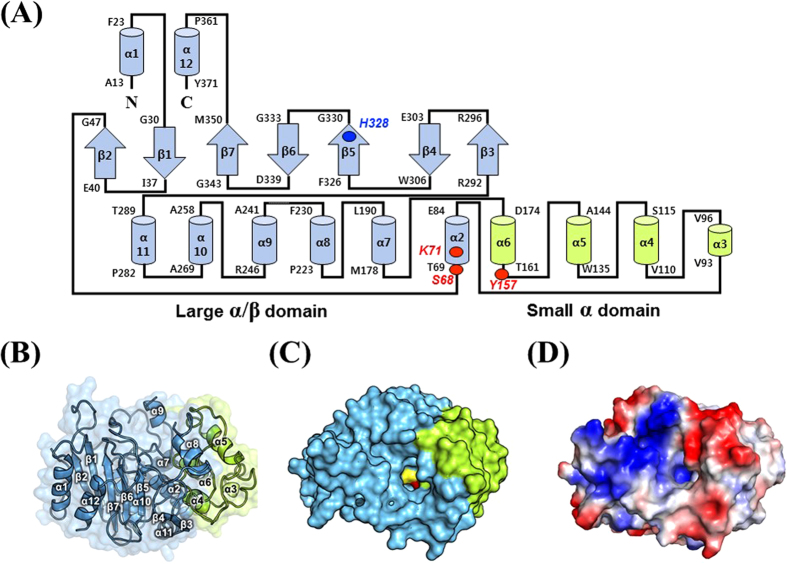
Structural visualization and topology diagram of CcEstA. The large α/β domain and small α domain of CcEstA are distinguished by different colors (light blue and light green, respectively). (**A**) Topology diagram of CcEstA. The catalytic triad residues are shown in red, and His^328^ in Motif III in blue. The dashed line between α8 and α9 represents a missing invisible region of electron density. (**B**) The overall structure of CcEstA is shown with a ribbon diagram. (**C**) Molecular surface of CcEstA. The substrate-binding pocket of CcEstA is shown at the center. Inside the pocket, the catalytic Ser and Tyr are shown in yellow and red, respectively. (**D**) Surface charge distribution of CcEstA. Blue and red regions indicate local positive and negative charge, respectively.

The crystal structure of CcEstA that belongs to the *P*2_1_2_1_2_1_ space group was determined at a 1.9-Å resolution with R_work_ of 17.04% (R_free_ = 20.10%). The crystallographic statistics for data collection and structure refinement are summarized in Table 1. Although most residues of CcEstA are well-defined, residues located in positions 237 to 239 were not visible in the final 2*F*_*o*_−*F*_*c*_ electron density map. In many PBP structures, disorders of similar loop regions are observed^32^,^33^. A Ramachandran plot revealed that all residues are in the most favored and additionally allowed regions (data not shown). The overall structure of CcEstA consisted of two domains: a large α/β domain and a small α domain (Fig. 8A), which is also found in other PBPs and serine β-lactamases^34^. The large α/β domain (residue 1–85 and 175–374) has a seven-stranded anti-parallel β-sheet (β2, β1, β7, β6, β5, β4, and β3) enclosed by six helices (α2, α7, α8, α9, α10, and α11) on one face and two helices (α1 and α12) on the other face (Fig. 8B). The small α domain (residue 86-174) includes four helices (α3, α4, α5, and α6). These two domains create a deep hydrophobic tunnel in which the catalytic serine is located 8.5 Å near the bottom of the cleft (Fig. 8C). The four residues (S^68^-T^69^-T^70^-K^71^) in Motif I are found at the N-terminal end of α2 helix. Tyr^157^ of Motif II lies on the loop between α5 and α6 helices. Histidine (His^328^) and glycine (Gly^330^) of Motif III are situated at the C-terminal end of β6. Figure 8D shows the electrostatic surface of CcEstA, highlighting the positive charges of many arginine and lysine residues present in the large α/β domain. Specifically, a cluster of aspartic acids (Asp^128^, Asp^134^, and Asp^136^) and glutamic acid (Glu^126^) are observed in the small α domain, which are highly conserved in other related PBPs.

**Figure 9:**
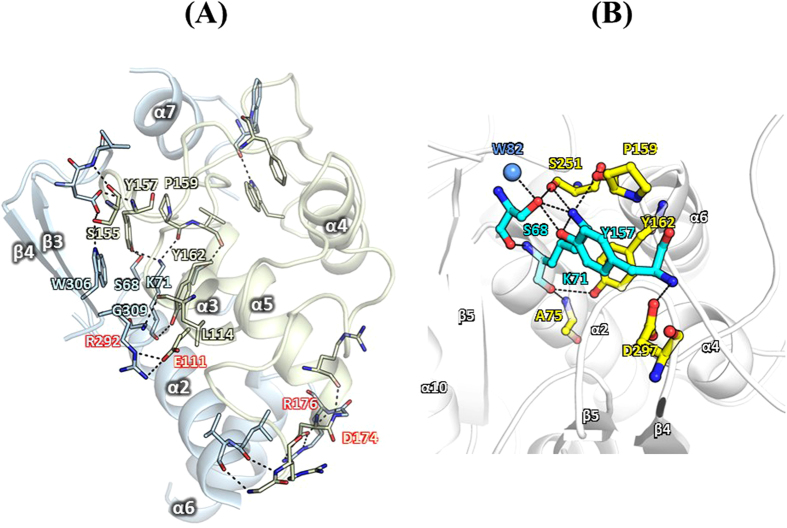
Domain-domain interfaces and hydrogen-bonding networks in the active site of CcEstA. (**A**) The interfaces between the large α/β domain (light blue) and the small α domain (light green) are shown. Electrostatic interactions and hydrogen bonds are shown with dashed lines. (**B**) The hydrogen-bonding network of CcEstA around the active site is shown.

The domain-domain interactions of CcEstA are mainly composed of residues from α2, α4, α6, and β4 with residues from three loops (α2/α3, α4/α5, α5/α6, α6/α7 loop, α8/α9 loop, and β4/β5 loop), which include salt-bridges of Arg^176^ (on the loop between α6 and α7) – Asp^174^ (on the loop between α6 and α7) and Arg^292^ (on β4) - Glu^111^ (on α4). Extensive hydrogen bonding networks were also formed including Lys^71^, Leu^81^, Val^82^, Gly^175^, Arg^176^, Trp^233^, Arg^292^, and Asp^297^, and Leu^298^ of the large α/β domain and Gly^85^, Arg^86^, Leu^87^, Glu^111^, Phe^123^, Trp^132^, Ser^155^, Tyr^157^, Pro^159^, Tyr^162^, Arg^171^, and Asp^174^ of the small α domain (Fig. 9A). The active site of CcEstA is located in the cleft at the interface between the α/β domain and the small α domain, which is lined by a series of highly conserved residues. The active site of CcEstA shows a compact hydrogen-bonding network around catalytic residues to activate Ser^68^ for the acylation step (Fig. 9B). Specifically, Lys^71^-Nζ forms hydrogen bonds with the Oγ of Ser^68^ (2.6 Å), Oη of Tyr^157^ (2.9 Å), and the carbonyl group of Pro^159^ (2.8 Å) and Ser^251^ (3.0 Å). An amide group of Lys^71^ is also involved in a hydrogen-bonding network with a carbonyl group of Ser^68^ and Oγ of Ser^68^ was found to be spatially separated from Oη of Tyr^157^ by 2.7 Å. Therefore, Oγ of Ser^68^ forms two hydrogen bonds to Nζ of Lys^71^ and Oγ of Ser^68^.

**Figure 10:**
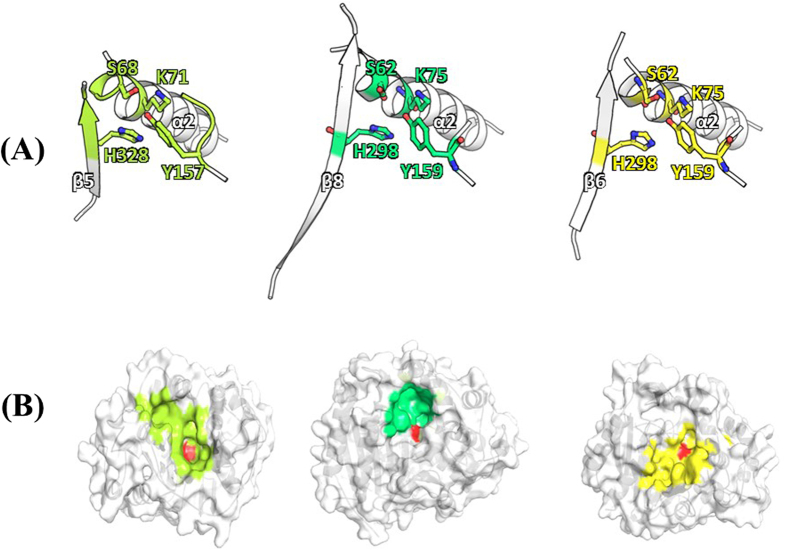
Comparison of the active site of CcEstA, R61 DD-peptidase (3PTE), and *Pa*PBP (2QMI). (**A**) Ribbon diagram of enzyme active sites. Key residues (Ser and Lys in motif I, Tyr in motif II, and Histidine in motif III) in the active site are highlighted and shown as ball-and-stick model. (**B**) Molecular surfaces of the enzyme active site. Nucleophilic Ser is shown in red and other highly conserved residues (Lys, Tyr, and His) in the same color scheme with (**A**).

To compare CcEstA to other homologous proteins, DALI and BLAST searches were executed with available structures in the Protein Data Bank (PDB). CcEstA showed structural similarity to R61 DD-peptidase (PDB ID: 3PTE, *Z*-score 28.8) from *Streptomyces* R61, with root-mean-square deviations (RMSD) of 2.68 Å for 279 Cα atoms. Furthermore, CcEstA also showed structural similarity to a PBP from *Pyrococcus abyssi (Pa*PBP, PDB ID:2QMI, *Z*-score 30.0), with an RMSD of 2.46 Å for 288 Cα atoms. These two enzymes have 15.2 and 12.6% sequence identity with CcEstA, respectively, and only 34 residues are completely conserved among these enzymes (Fig. S1). The structural comparison of CcEstA with R61 DD-peptidase and *Pa*PBP revealed that catalytic residues are completely conserved among these enzymes. Locations of Ser^68^, Lys^71^, Tyr^157^, and His^328^ in CcEstA are structurally equivalent to those of R61 DD-peptidase and *Pa*PBP (Fig. 10A). Furthermore, the spatial locations of glycine in Motif III of CcEstA (Gly^330^), involved in oxyanion hole formation, are similar to those of R61 DD-peptidase (Gly^331^) and *Pa*PBP (Gly^337^) (Fig. S1). In addition, the area of domain-domain interface of CcEstA (1720 Å^2^) is similar to that of *Pa*PBP (1778 Å^2^), but larger than that of R61 DD-peptidase (1406 Å^2^). Interestingly, significant differences were observed in the size and shape of their substrate-binding pockets (Fig. 10B).

### Substrate-binding pocket of CcEstA

**Figure 11:**
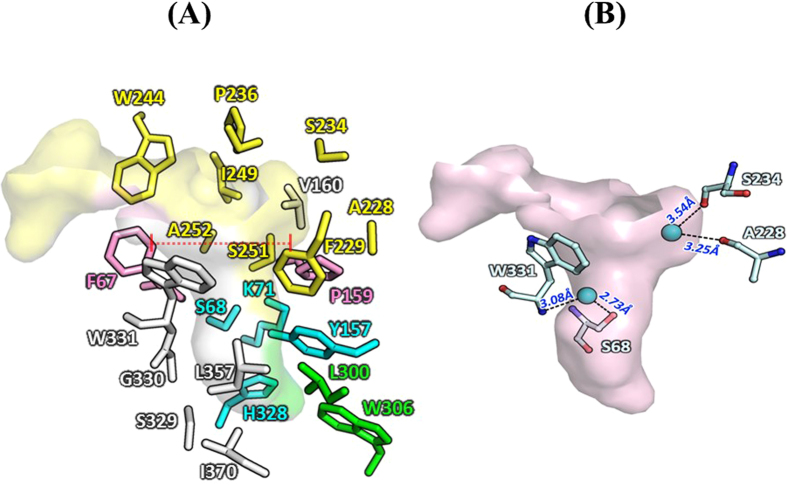
Substrate-binding pocket of CcEstA. (**A**) All residues forming the pocket are shown as a ball-and-stick model. Key residues of the active site are colored in cyan, the residues of UL in yellow, LL in green, and others in white. Two bulky residues of F67 and P159 are shown in purple and roof regions were indicated as a dotted line. (**B**) Four residues with two water molecules around the pocket of CcEstA are shown.

In previous reports, size and shape of the substrate-binding pocket are shown to affect substrate specificity of PBPs^20^, DD-peptidase^35^, and D-amino acid amidase^21^. Using CASTp server, the substrate-binding pocket of CcEstA was compared to that of homologous proteins. Interestingly, CcEstA showed a moderate pocket area (371.2 Å^2^) and volume (267.8 Å^3^) comparable to those of R61 DD-peptidase (203.80 Å^2^, 229.0 Å^2^). However, the pocket area and volume of *Pa*PBP (1184.5 Å^2^, 1963.7 Å^2^) are relatively larger than the other two enzymes. In CcEstA, the upper end of the substrate-binding pocket is delineated by α8, α9, α8/α9, and α9/α10 including Val^160^, Ser^234^, Ser^235^, Pro^236^, and Trp^244^, Ile^249^, Ser^251^, and Ala^252^, with the side chains of Phe^67^ and Pro^159^ forming a roof over the cleft (Fig. 11A). The Gly^327^-His^328^-Ser^329^-Gly^330^ motif was attached to the side end of the cleft through a short strand β5, and the indole ring of Trp^331^ was inserted into the active site. The α5/α6 and β3/β4 loops as well as Leu^300^ and Trp^306^ lay along one side of the substrate-binding cleft to form a wall with the β5/β6 loop forming the opposite wall. Furthermore, Ser^251^ and Ala^252^ together form part of the substrate-binding pocket of CcEstA and Pro^159^, Val^160^, Ile^249^, His^328^, and Ser^329^ are involved in the hydrogen-bonding network of this region. As shown in Fig. 11A, Phe^67^ and Trp^331^ take one side of the wall with a relatively large surface area and Tyr^157^and Phe^229^ take possession of the other side of the substrate-binding pocket. A hydrophobic region in the bottom side of the pocket was maintained by Leu^300^, Trp^306^, Met^311^, His^328^, Ser^329^, Gly^330^, Leu^357^, and Ile^358^. A small β5 strand having a conserved H^328^-S^329^-G^330^ motif (Motif III) forms the lower end of the cleft. Two water molecules (W1 and W2) were observed in the substrate-binding pocket of CcEstA (Fig. 11B). A water molecule (W1) is anchored in the pocket by hydrogen-bonding interactions with Ser^68^ and the main-chain nitrogen of Trp^331^ from Motif III, which seems to be necessary for the efficient deacylation of substrates. Another water molecule (W2) at the other side of the catalytic residues stabilizes the α8 helix through hydrogen bonds with Ala^228^ and Ser^234^. Similar roles of water molecules are also observed in OXA-58 Class D β-lactamase^36^.

**Figure 12:**
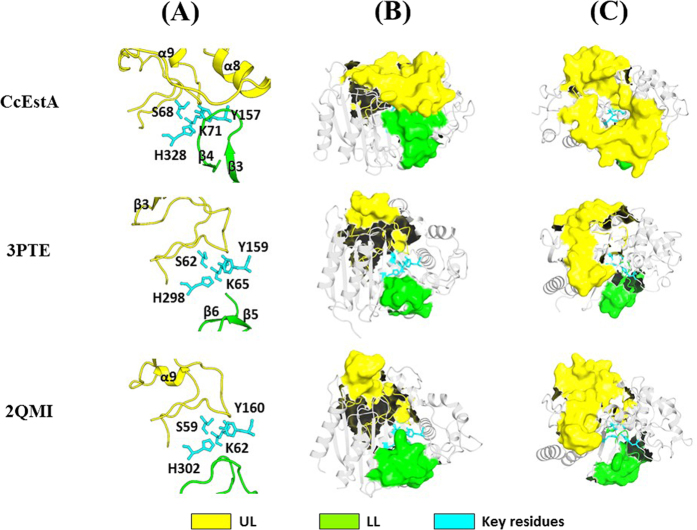
Analysis of UL and LL in CcEstA, R61 DD-peptidase (3PTE), and *Pa*PBP (2QMI). (**A**) Catalytic residues are colored in cyan. The residues of UL are in yellow and those of LL are in green. (**B,C**) Molecular surface representation of UL and LL in ribbon diagram. Front view (**B**) and top view (**C**) are shown. Note that the orientation of UL and LL in CcEstA is different from those of R61 DD-peptidase (3PTE) and *Pa*PBP (2QMI).

As shown in Fig. 12, two extensive stretches of upper loop (UL: Gly^191^–Thr^257^) and lower loop (LL: Ala^290^–Ala^308^) over the active site constitute the entrance region for the substrate-binding pocket. The *B*-factor values of these regions were remarkably high compared to those of other regions, indicating that the conformational flexibility of these regions may be important for substrate binding. Specifically, *B*-factor values for this region of CcEstA were 26.7 Å^2^ (UL) and 22.1 Å^2^ (LL) for the main chain, and 27.4 Å^2^ (UL) and 27.4 Å^2^ (LL) for side chains, which are larger than the average *B*-factors of main-chain atoms (22.3 Å^2^) and side-chain atoms (22.1 Å^2^). In addition, the upper loop region (α8/α9 loop), flanked by α8 and α9 in CcEstA, makes few contacts with the rest of the protein, which may explain the conformational flexibility that is necessary for substrate binding and structural reorganization. The lower loop region of the β3/β4 loop, which projects towards the α7/α8 loop of the upper loop, wraps around catalytic residues and partially forms part of the substrate-binding site. However, in R61 DD-peptidase, the corresponding loop folds away from the active site, which enables large substrates to come into the substrate-binding pocket. Furthermore, the corresponding region in *Pa*PBP is fully made up of flexible loops without any intervening β-strands. Therefore, accessibility toward the substrate-binding pocket of CcEstA is significantly more restricted than in R61 DD-peptidase and *Pa*PBP. Differences in substrate specificity among these enzymes can be explained on the basis of their structural differences in this region. Interestingly, PBPs are known to have the ability to make large structural changes from quantum mechanics/molecular mechanics calculations^37^ or structural and functional analysis^38^,^39^,^40^,^41^. In accordance, changes in length, sequence, and conformation of loops around active sites are supposed to play a key role in the divergence of enzyme function^42^,^43^.

In summary, a novel paralog (CcEstA) of PBPs from *Caulobacter crescentus* CB15 was characterized using biochemical analysis including native-PAGE, mass spectrometry, gel filtration, CD spectroscopy, fluorescence, reaction kinetics, and enzyme. CcEstA was shown to hydrolyze preferentially short-chain p-nitrophenyl esters, naphthyl esters, tertiary alcohol esters, and β-lactam nitrocefin. In addition, CcEstA structure revealed the flexibility in the substrate-binding pocket with two large loops of UL and LL. Structural and functional features of CcEstA derived from this analysis will provide a starting point for a comprehensive understanding of catalytic and structural features of PBP family and for the design of a novel class of antibiotics targeting PBP families. Although the role of CcEstA in peptidoglycan biosynthesis needs still to be explored, this protein might be involved in the remodeling of peptidoglycan structure with its hydrolyzing activity. Further crystallographic analysis of CcEstA complexed with chemical ligands, mutagenesis of key residues, and in-depth kinetic analysis are necessary for a thorough understanding the substrate recognition mechanism of this enzyme.

## Materials and Methods

### Gene cloning and protein purification

Cloning and expression of CcEstA were performed as previously described^17^. Briefly, the CcEstA gene was PCR-amplified from chromosomal DNA of *C. Crescentus* CB15 (Microbank of Microbial Genomics and Application Center, Daejon, Republic of Korea). The resulting PCR product was inserted into a pQE30 vector, where 12 additional codons encoding a His-tag (MRGSHHHHHHGS) were placed in frame at the N-terminal region. The construct was overexpressed in *Escherichia coli* XL1-Blue cells upon induction with 1 mM IPTG at 37 °C for 4 h. The harvested cells were lysed by sonication in lysis buffer (20 mM Tris-HCl pH 8.0, 100 mM NaCl). The lysate was centrifuged and the supernatant was loaded onto a His-Trap column (GE Healthcare). The column was washed with 5 column volumes of lysis buffer containing 40 mM imidazole. The protein was then eluted with lysis buffer containing 200 mM imidazole and desalted on a PD-10 column (GE Healthcare) with 50 mM Tris-HCl pH 8.0. The purified CcEstA was concentrated to 1 mg/mL in 50 mM Tris-HCl pH 8.0 using Vivaspin concentrators (GE Healthcare) without cleaving the N-terminal His-tag. The purity of the CcEstA was confirmed by SDS-PAGE and protein concentration was determined by the Bradford method (Bio-Rad, USA).

### Biochemical assays of CcEstA

The esterase activity of CcEstA was determined mainly with *p*-nitrophenyl esters by measuring the absorbance at 405 nm using an EPOCH2 microplate reader (Biotek), as described previously^17^. The standard assay solution consisted of 250 μM substrate solution in 20 mM Tris-HCl pH 8.0 with 10 μg CcEstA protein (at a final concentration of ~800 nM). The naphthyl-group derivatives 1-naphthyl acetate (1-NA), 1-naphthyl butyrate (1-NB), 2-naphthyl acetate (2-NA), and 1-naphthyl phosphate (1-NP) were also used as substrates for analyzing regioselectivity, and the absorbance was determined at 310 nm under the same condition as described above.

Thermostability of CcEstA was measured by incubating the enzyme at 20 °C, 30 °C, 40 °C, 50 °C, and 60 °C for 1 h. Then, each aliquot was properly taken every 15 min for measuring the residual activity. The pH stability of CcEstA was examined by measuring catalytic activity in the pH range of 4.0–11.0. The buffer systems included citrate-NaOH (pH 3.0–6.0), phosphate-NaOH (pH 7.0), Tris-HCl (pH 8.0), and glycine–NaOH buffer (pH 9.0–11.0). To examine the effects of chemicals, CcEstA was incubated with each compound for 1 h at 25 °C, and the residual activity was determined using 10 mM *p*-nitrophenyl acetate (pNA) as a substrate.

For enantioselectivity analysis, CcEstA was incubated with either (*R*)- or (*S*)-solutions^44^. The (*R*)- and (*S*)-solutions consisted of phenol red (1 mg/mL), 20 mM Tris-HCl pH 8.0 and 300 mM methyl-(*R*)-(−)-3-hydroxy-2-methylpropionate or methyl-(*S*)-(+)-3-hydroxy-2-methylpropionate solution. This pH indicator-based colorimetric assay was also performed to verify the activity of CcEstA toward cefotaxime (CTX) and 7-aminocephalosporanic acid (7-ACA), respectively. CcEstA was incubated with 12 mM CTX or 7-ACA at 25 °C for 1 h. The β-lactamase activity of CcEstA was determined using a chromogenic β-lactam substrate, nitrocefin [2 *S*,5 *R*,6 *R*)-6-{[carboxy(phenyl)acetyl]amino}-3,3-dimethyl-7-oxo-4-thia-1-azabicyclo[3.2.0] heptane-2-carboxylic acid]. CcEstA (0.5 mg/ml) was reacted with a 484 μM nitrocefin solution in 20 mM Tris-HCl pH 8.0 at 25 °C, and the color change in the reaction mixture was observed.

### Mass spectrometry, CD spectroscopy, and fluorescence analysis

Matrix-assisted laser desorption/ionization time-of-flight mass spectrometry was performed with a Voyager DE STR system in positive-ion mode^44^. For an overlay activity assay, native-PAGE was performed and the gel was rinsed several times with 50 mM sodium phosphate buffer at pH 7.0. Then, 0.25 mM 4-methylumbelliferyl (4-MU) acetate was added to detect blue fluorescence under UV illumination, which is emitted from 4-MU after hydrolysis^45^. The molecular mass of CcEstA was estimated by gel filtration FPLC (AKTA UPC-900, GE Healthcare) on a Superdex 200GL column (GE Healthcare).

Circular dichroism (CD) spectra were recorded with a Jasco J715 spectropolarimeter with a Peltier temperature control (Jasco, MD, U.S.A.) Far-UV CD analysis were carried out in the wavelength range of 190 to 260 nm with a 0.5 nm bandwidth, 1 s response time, and 200 nm/min scan speed. All spectra were corrected by the subtraction of the buffer signal. The raw CD signal, θ, was converted to mean residue ellipticity using the equation [θ]_MRW_ = θ/(10*C*_r_*l*), where *C*_r_ represents the concentration and *l* is the path length. For thermal unfolding analysis, the CD signal was monitored at 222 nm using a 0.1 cm path length at a scan rate of 1 °C/min with 1 mg/ml CcEstA.

All fluorescence measurements were carried out using a Jasco FP-6200 spectrofluorometer (Jasco, MD, U.S.A.). Urea-induced unfolding of CcEstA was performed by incubating the protein with various concentrations of urea at 25 °C for 1 h. Protein samples were excited at 290 nm, and the emission spectra were recorded in the range of 300 to 400 nm. All fluorescence spectra were recorded with a scan speed of 250 nm/min with a 5 nm slit width.

### Kinetic assays

Kinetic assays were performed using *p*-NA, *p*-NO, and nitrocefin as substrates. Enzyme solutions were prepared in 20 mM Tris-HCl pH 8.0 buffer. The enzyme concentrations differed between *p*-nitrophenyl esters and nitrocefin reactions (40 nM or 15 μM of CcEstA, respectively). Various concentrations of substrate were added to the enzyme solutions. The reactions were performed at RT, and monitored using the EPOCH2 microplate reader (Biotek) at 405 nm for *p*-nitrophenyl esters, and at 486 nm for nitrocefin. Measurements were taken every 10 s for 15 min (1 min for 5 h with nitrocefin). Each concentration of substrate was measured along with a blank and at least 3 measurements were collected to determine the rate. Sample path-length correction was done automatically by Gen5 software (Biotek, USA). The molar extinction coefficients of *p*-nitrophenol and nitrocenfin were determined experimentally (*p*-nitrophenol: 17,154 M^−1^ cm^−1^, and nitrocefin: 12,124 M^−1^ cm^−1^). The reaction velocities were determined from the initial linear portion of the progress curves, which were generated after subtracting absorbance of the blank, correcting sample path-length, and converting absorbance into product concentration [P] with the experimentally obtained extinction coefficient. From the *x-* and *y-*intercepts of the Lineweaver-Burk plot, parameters of *V*_max_ and *K*_m_ were obtained for each substrate. The *k*_cat_ was calculated using the equation *k*_cat_ = *V*_max_/[E]_t_.

### Crystallization and X-ray Data collection

Crystallization was performed using the microbatch crystallization method under a thin layer of Al’s oil, using the commercially available screening kits Wizard I, II (Emerald BioSystems, Washington, USA) and Crystal Screen I, II (Hampton Research, CA, USA) at 295 K. Diffraction-quality crystals were obtained from the condition Wizard I-34 (1.0 M ammonium phosphate dibasic, 0.1 M imidazole pH 8.0) and Crystal Screen I-42 (50 mM potassium phosphate monobasic, 20% w/v polyethylene glycol 8000). Crystals were immediately frozen in a cold nitrogen stream for MAD data collection. For X-ray data collection, the CcEstA crystal was transferred in the cryosolvent containing the same reservoir and 20% glycerol before being flash-frozen in a cold nitrogen stream. X-ray diffraction data were collected at 100 K using an ADSC Quantum 315 CCD on beamline PAL 4 A at the Pohang Accelerator Laboratory (PAL), Korea. The data were indexed, integrated and scaled using HKL-2000. The data collection and processing statistics are summarized in Table 1.

### Structure determination and refinement

The structure of CcEstA was solved by MOLREP^46^ using the molecular replacement method using the crystal structure of EstA from *Arthrobacter nitroguajacolicus* Rue61a (PDB 3ZYT) as a template. The automated model building was performed by PHENIX Autobuild. The model was refined many steps using REFMAC^47^ and PHENIX^48^. The quality of the final model was evaluated with the program MOLPROBITY^49^. Refinement statistics are given in the Table 1.

### Sequence and structure analysis

To identify related enzymes in the PBP family, protein data bank (PDB) was searched for sequences similar to the primary sequence of CcEstA. All primary sequences were retrieved from Swiss-Prot in FASTA format^50^. Multiple sequence alignments were performed using Clustal Omega^51^ and the results were rendered using ESPript^52^. The enzymes possessing structural similarity to CcEstA were found using the DALI server^53^. Two related proteins (R61 DD-peptidase from *Streptomyces* R61 and *Pa*PBP from *Pyrococcus abyssi*) that were found in both BLAST and DALI searches, were selected for structural comparison with CcEstA. Superposition of the enzymes was performed using CCP4 software^54^. *PyMOL* v1.8 was used to represent three-dimensional structures of the enzymes (PyMOL Molecular Graphics System, Schrödinger, LLC). Coordinates and structure factors have been deposited in the RCSB Protein Data Bank as accession number 5GKV.

## Additional Information

**Accession codes:** The coordinates and structure factors for CcEstA have been deposited in the Protein Data Bank with code: 5GKV.

**How to cite this article**: Ryu, B. H. *et al*. Biochemical and Structural Analysis of a Novel Esterase from *Caulobacter crescentus* related to Penicillin-Binding Protein (PBP). *Sci. Rep.* **6**, 37978; doi: 10.1038/srep37978 (2016).

**Publisher's note:** Springer Nature remains neutral with regard to jurisdictional claims in published maps and institutional affiliations.

## Supplementary Material

Supplementary Information

## Figures and Tables

**Table 1:** Data collection and refinement statistics for CcEstA structure.

	EstCC
Data collection	
Space group	*P*2_1_2_1_2_1_
Cell dimensions	
*a, b, c* (Å)	58.277
	67.308
	92.093
α, β, γ (°)	90, 90, 90
Molecules/AU	1
Wavelength	0.919805
Resolution (Å)	50–1.90
No. reflections	29140
*R*_sym_ or *R*_merge_^1^	10.7 (37.9)
Mean (*I*/σ*I)*	34.8 (5.3)
Completeness (%)	99.9 (100)
Redundancy	7.2 (7.3)
Refinement	
Resolution (Å)	30–1.9
No. reflections	28932
*R*_work_^2^/*R*_free_^3^	17.04/20.10
No. atoms	
Protein	2801
Ligand/ion	—
Water	348
*B*-factors	
Protein	21.24
Ligand/ion	—
R.M.S.D^4^	
Bond lengths (Å)	0.007
Bond angles (°)	0.857
Ramachandran statistics^5^ (%)	97.28/2.72/0.00
Completeness (%)	99.9 (100)

(Values in parentheses refer to the last resolution shell).

^1^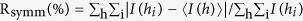, where *I(h*_*i*_) is the single intensity of reflection *h* as determined by the *i*th measurement and <*I(h*)> is the mean intensity of reflections *h*.

^2^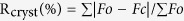, where, *Fo* is the observed structure factor amplitude, and *Fc* is the structure factor calculated from the model.

^3^R_free_ (%) is calculated in the same manner as R_cryst_ using 5% of all reflections excluded from refinement stages using high resolution data.

^4^R.M.S.D., Root-mean-square deviation.

^5^The fraction of residues in the favored, allowed, and disallowed regions of the Ramachandran diagram calculated by MOLPROBITY.
