# Supplementary material for: Biochemical and Structural Analysis of a Novel Esterase from Caulobacter crescentus related to Penicillin-Binding Protein (PBP)
Source: Sci Rep. 2016 Dec 1;6:37978. doi: 10.1038/srep37978 (PMC5131357; doi:10.1038/srep37978)
Supplement: Supplementary Information [file srep37978-s1.pdf]

Biochemical and Structural Analysis of a Novel Esterase from *Caulobacter crescentus* related to Penicillin-Binding Protein (PBP)

Bum Han Ryu[1,2⊥], Tri Duc Ngo[2⊥], Wan Ki Yoo[1,2], Sojeong Lee[1], Boo-Young Kim[1], Euijoo Lee[1], Kyeong Kyu Kim[2]*, T. Doohun Kim[1]*

[1]Department of Chemistry, College of Natural Science, Sookmyung Women's University, Seoul 140-742, Korea; [2]Department of Molecular Cell Biology, Samsung Biomedical Research Institute, Sungkyunkwan University School of Medicine, Suwon, 440-746, Korea

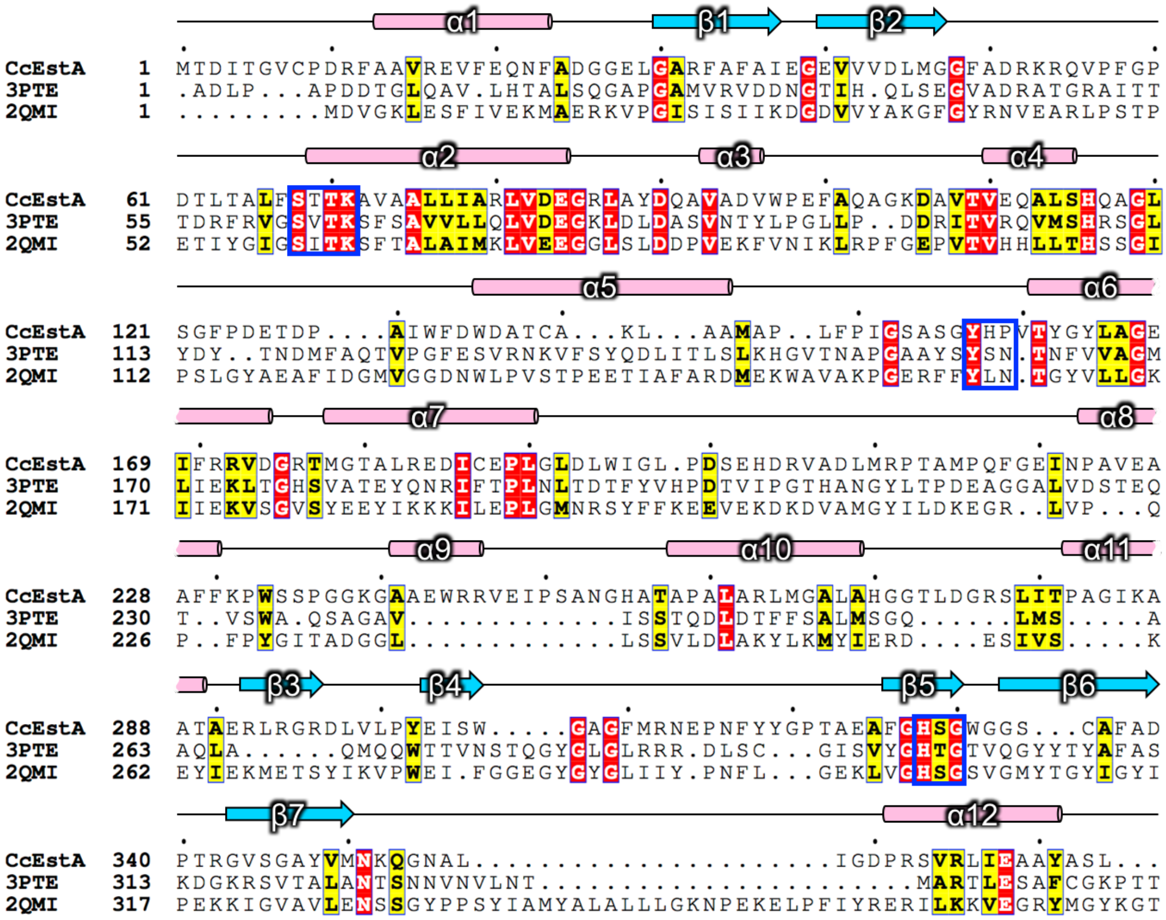

Figure S1. Multiple sequence alignment of CcEstA, R61 DD-peptidase (3PTE), and *Pa*PBP (2QMI). Highly conserved residues are shown in red and three common motifs (Motif I, II, and III) are shown in blue boxes.

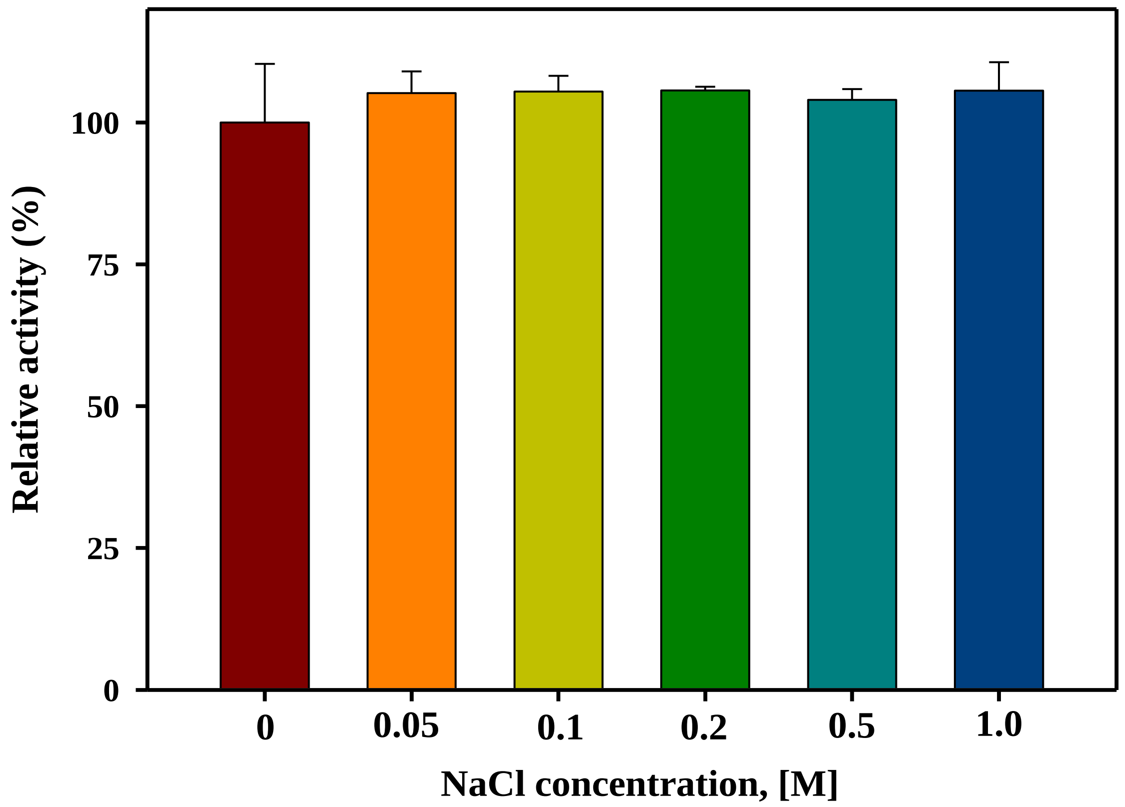

Figure S2. Activity of CcEstA in the presence of various NaCl concentrations (0 - 1.0 M). Please note that addition of NaCl showed almost no activity loss of CcEstA.

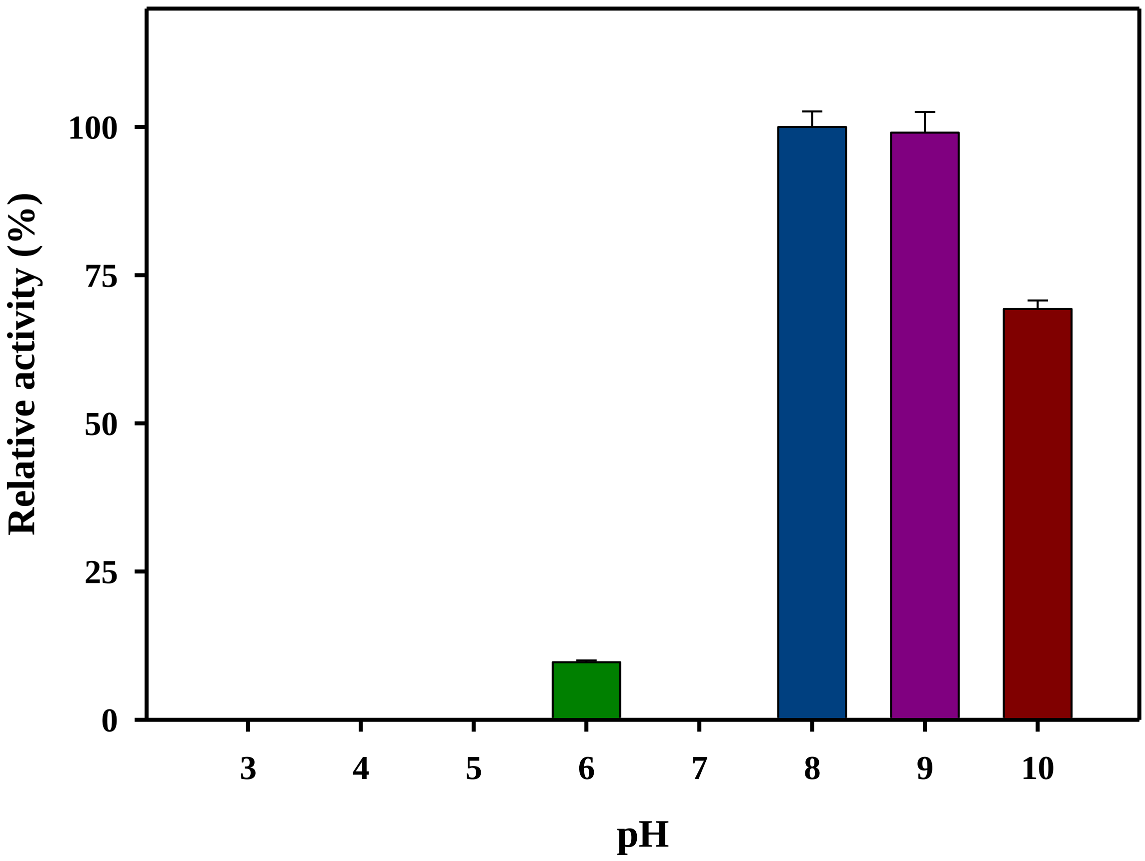

Figure S3. Activity of CcEstA in different pH solutions from pH 3 to 10. The buffer systems included malic acid buffer (pH 3.0), sodium acetate (pH 4.0-5.0), sodium cacodylate (pH 6.0), Imidazole (pH 7.0), HEPES(4-(2-hydroxyethyl)-1-piperazineethanesulfonic acid)-KOH(pH 8.0), and sodium borate (pH 9.0–10.0).

/

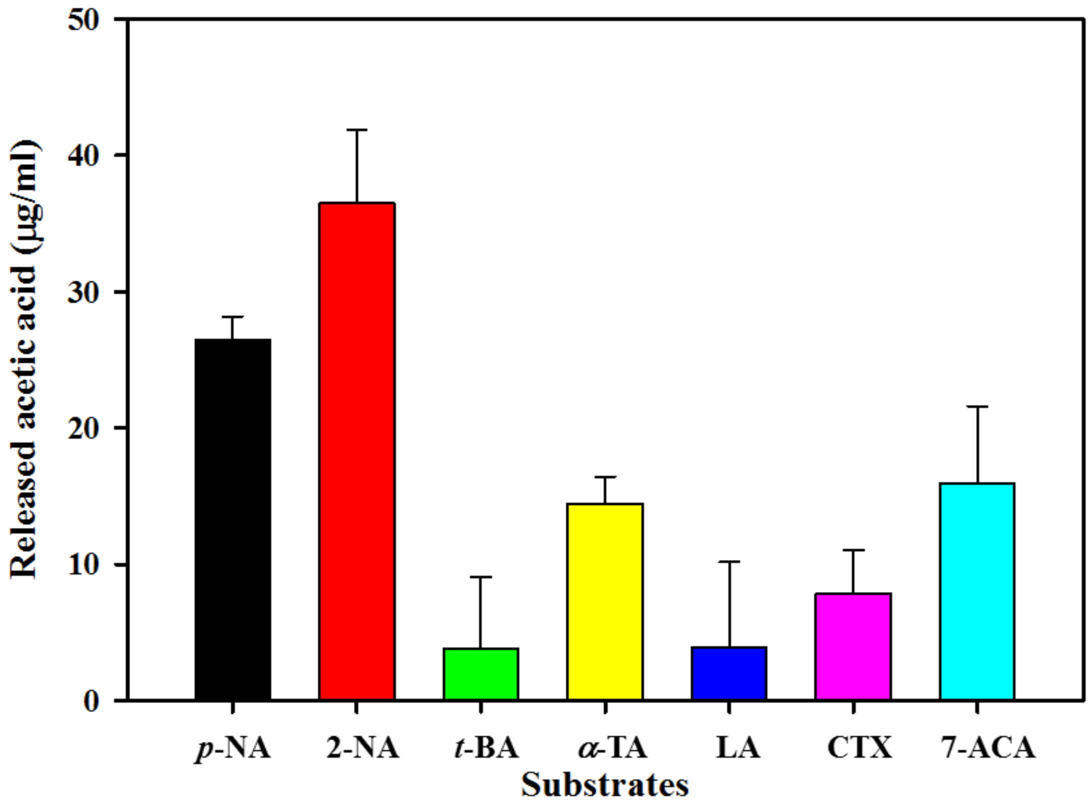

Figure S4. Deacetylation activity of CcEstA. The amount of released acetic acids from various substrates (250 μM) was quantitatively determined using acetic acid determination kit (Megazyme Inc., Chicago, IL, USA). The highest activities were obtained with 2-naphthyl acetate (2-NA), followed by *p*-nitrophenyl acetate (*p*-NA), and 7-aminocephalosporanic acid (7-ACA). Other substrates including *tert*-butyl acetate (t-BA), α-terpinyl acetate (α-TA), linalyl acetate (LA), and cefotaxime (CTX) were also used for comparison.
